# Perceived ability to regulate love

**DOI:** 10.1371/journal.pone.0216523

**Published:** 2019-05-13

**Authors:** Kruti Surti, Sandra J. E. Langeslag

**Affiliations:** Department of Psychological Sciences, University of Missouri–St. Louis, St. Louis, MO, United States of America; UNSW Sydney, AUSTRALIA

## Abstract

Research has shown that romantic love can be regulated. We investigated perceptions about love regulation, because these perceptions may impact mental health and influence love regulation application. Two-hundred eighty-six participants completed a series of items online via Qualtrics that assessed perceived ability to up- and down-regulate, exaggerate and suppress the expression of, and start and stop different love types. We also tested individual differences in perceived love regulation ability. Participants thought that they could up- but not down-regulate love in general and that they could up-regulate love in general more than down-regulate it. Participants thought that they could up-regulate infatuation less than attachment and sexual desire. Participants also thought that they could exaggerate and suppress expressions of infatuation, attachment, and sexual desire, but that they could not start and stop infatuation and attachment, or start sexual desire. The more participants habitually used cognitive reappraisal, the more they thought that they could up- and down-regulate infatuation and attachment and up-regulate sexual desire. The more participants were infatuated with their beloved, the more they thought that they could up- but not down-regulate infatuation, attachment, and sexual desire. Finally, participants thought that they could up- and down-regulate happiness more than infatuation These findings are a first step toward the development of psychoeducation techniques to correct inaccurate love regulation perceptions, which may improve mental health and love regulation in daily life.

## Introduction

Do you think you can regulate how in love you are? Love can be weaker than desired. For example, studies have shown that love declines over time [1] and that falling out of love is one of the main reasons for divorce [2]. Love can also be stronger than desired, such as after a break-up. In these types of situations, it might be advantageous to regulate love. Love regulation is the use of behavioral and cognitive strategies to change the intensity of love [3]. Research has shown that love regulation is feasible [3–9], but research on people’s perceptions about love regulation (i.e., whether they think that love can be regulated) is scarce.

It is well-known that negative and positive emotions can be both up- and down-regulated using regulation strategies such as distraction, cognitive reappraisal, and expressive suppression [10]. Cognitive reappraisal involves changing the interpretation of a situation to change the intensity of the emotion. It is considered an effective and adaptive regulation strategy. Expressive suppression involves inhibiting expressions of emotion. Although it changes the way emotions are expressed, it does not reduce the emotional experience and can have negative cognitive and social consequences [11]. Even though there is an ongoing debate regarding whether love is an emotion or not [12–18], previous studies have shown that love can be regulated using emotion regulation strategies such as distraction and cognitive reappraisal [3, 7, 9, 19].

There are different types of love, including but not limited to compassionate love, maternal love, infatuation, attachment, and sexual desire [20–26]. For the purpose of this study, we focus on the latter three types, as those are types of romantic love. Infatuation (or passionate love) is the intense amorous feeling towards a person that arises initially when one falls in love, attachment (or companionate love) is characterized by the formation of an emotional bond with the person, and sexual desire (or lust) is a craving for sexual gratification [22–25, 27, 28]. Previous studies have shown that infatuation and attachment were somewhat negatively correlated [29]. In addition, infatuation is often, but not necessarily, accompanied by sexual desire [20].

Multiple studies have shown that love can be regulated. In one study, thinking negatively about an ex-partner decreased self-reported levels of love in general for that ex-partner [9]. In another study, thinking about negative aspects of the beloved or the relationship, or imagining negative future scenarios (i.e., negative reappraisal) decreased both infatuation and attachment, and thinking about positive aspects of the beloved or the relationship, or imagining positive future scenarios (i.e., positive reappraisal) increased attachment [3]. Likewise, people with sexual dysfunctions could up-regulate sexual desire by imagining or fantasizing about sexual situations, or by focusing on positive aspects of their body [7]. Another study showed that when men were instructed to inhibit emotional reactions to an erotic film they reported lower levels of sexual arousal than when they were instructed to allow themselves to become sexually aroused [6]. Additionally, women who were instructed to maximize sexual arousal during an erotic film had greater self-reported and genital arousal than women who were instructed to suppress sexual arousal [5]. In short, people can successfully regulate infatuation, attachment, and sexual desire using a variety of regulation strategies.

Now that it is clear that love regulation is feasible, it is crucial to study people’s perceptions about love regulation for two reasons. First, whether people feel in control or not affects their mental and physical well-being. For example, it has been shown that perceived lack of control over anxiety-related events is positively associated with self-reported anxiety levels in teens [30]. Also, perceiving emotions as uncontrollable is associated with lower well-being [31] and higher levels of pathological distress [32]. So, incorrect perceptions that love cannot be regulated may have negative consequences for well-being. Second, whether people feel in control or not affects whether they exert control. That is, people’s beliefs about emotion influence each step of the emotion regulation process: identifying a need to regulate, selecting regulation strategies, implementing regulation, and monitoring regulation success [33]. Specifically, perceiving emotions as uncontrollable might be associated with less active emotion regulation efforts, reduced motivation to engage in active regulatory efforts, and less effort expended on regulating emotions [32]. In addition, perceived lack of control over emotions has been associated with increased intention to use avoidance strategies [34]. Crucially, the less people believed they could control emotions, the less successful they were in regulating their emotions [35, 36]. Given that love can be regulated using emotion regulation strategies [3, 7, 9, 19], the same principles may hold for love regulation. Specifically, any perceptions that love cannot be regulated might keep people from applying love regulation in daily life. Ultimately, knowledge of perceptions about love regulation could lead to psychoeducation to correct any incorrect perceptions that love cannot be regulated.

Perceptions about the feasibility of love regulation were studied in three previous studies only. In contrast to the evidence that love can be regulated, participants in one study reported that love in general is involuntary and uncontrollable [37] and participants in another study thought that love in general is somewhat uncontrollable [3]. Participants in the third study thought that down-regulation of love in general is neither possible nor impossible [9]. These previous studies suggest that incorrect perceptions about love regulation exist, which highlights the importance for further study of this topic. Although previous data suggest that people may be more successful at love down- than up-regulation [3], the previous studies about perceptions unfortunately did not test whether people’s perceptions about love regulation feasibility differed between up- and down-regulation. Therefore, the first goal of the current study was to investigate whether people think they can up- and down-regulate love in general and whether the perceived ability to regulate love in general differs between up- and down-regulation. For this first goal we focused on love in general to facilitate comparison to the previous findings discussed above [3, 9, 37]. Based on those findings, we expected people to think that they cannot up- or down-regulate love in general (hypothesis 1a). In addition, if people’s perceptions about love regulation align with regulation ability [3], we expected people to think that they can up-regulate love in general even less than down-regulate love in general (hypothesis 1b).

However, because there are different types of love, it is important to distinguish between them. Previous studies have shown that regulation of attachment was perceived as more feasible than regulation of infatuation [3, 9] and that people perceived more control over sexual than non-sexual (e.g., about dirt, disease, and contamination) intrusive thoughts [4]. Unfortunately, no previous studies compared the three different love types (i.e., infatuation, attachment, and sexual desire). Therefore, the second goal was to test whether people think they can up- and down-regulate infatuation, attachment, and sexual desire, and whether people’s perceived ability to up- and down-regulate differs between infatuation, attachment, and sexual desire. Based on previous studies regarding love in general [3, 9, 37], we expected people to think that they cannot up- or down-regulate infatuation, attachment, and sexual desire (hypothesis 2a). Also, based on the previous studies regarding actual ability to regulate different love types [3, 4, 9], we expected people to think that they can up- and down-regulate infatuation least (hypothesis 2b).

Research has shown that people can regulate the way they express emotions. For example, participants who were instructed to exaggerate their emotional reactions while viewing amusing or disgusting film clips displayed more valence and arousal in their facial expressions than participants who were instructed to view the film clips naturally [38]. In another study, participants who were instructed to suppress their emotional expressions while viewing amusing, sad, and neutral film clips showed less expressive behavior (such as facial expressions and body movements) than participants who received no suppression instructions [39]. Love is expressed through head nods, Duchenne smiles, gesticulation, forward leans, blushing, and pupil dilation [40, 41] and people might be able to exaggerate or suppress those expressions. To our knowledge, it has not been examined yet whether people can regulate love expression, and what their perceptions about that are. Therefore, the third goal was to investigate whether people think they can exaggerate and suppress expressions of infatuation, attachment, and sexual desire. Based on the previous studies [38, 39], we expected people to think that they can exaggerate and suppress expressions of infatuation, attachment, and sexual desire (hypothesis 3).

Furthermore, people are capable of initiating new emotions [42]. For example, participants were able to produce emotions such as anger by imagining a specific scenario [43]. People may want to start love from scratch in some situations. In an arranged marriage, for example, it may be beneficial to generate love towards the new spouse. Conversely, there are situations in which people may want to stop love completely, such as after a romantic break-up, or when people who are happily married develop an unwanted crush on someone else, or when people love someone they are not arranged to be married with. In a previous study, participants felt more control over the intensity of love than over who they are in love with [3]. This implies that people think they cannot start love from scratch or stop love completely, but that has not been tested directly. Therefore, the fourth goal was to investigate whether people think they can start and stop infatuation, attachment, and sexual desire. Based on the previous study [3], we expected people to think that they cannot start and stop infatuation, attachment, and sexual desire (hypothesis 4).

Because people may differ in beliefs about love regulation, the fifth goal was to investigate individual differences in the perceived ability to up- and down-regulate infatuation, attachment, and sexual desire. To start with, the previous study showed that the more people habitually used cognitive reappraisal, the more they felt they could control the intensity of love [3]. However, that study did not distinguish between perceptions about up- and down-regulation. We expected that the more people habitually use cognitive reappraisal, the more they think they can up- and down-regulate infatuation, attachment, and sexual desire (hypothesis 5a). Furthermore, we expected that the more people habitually use expressive suppression, the more they think they can suppress expressions of infatuation, attachment, and sexual desire (hypothesis 5b). Additionally, it could be that the perceived ability to regulate love is affected by whether someone is in love or not. In previous studies about love regulation perceptions, most if not all participants were in love because they were either in a relationship or had recently experienced a romantic break-up [3, 9]. Because emotion regulation requires cognitive control [44] and because love can be regulated using similar regulation strategies [3, 7, 9, 19], love regulation will probably require cognitive control as well. But because it has been suggested that love is associated with reduced cognitive control [45], we expected that people who are in love think they can up- and down-regulate infatuation, attachment, and sexual desire less than people who are not in love (hypothesis 5c). For the same reason, we also expected that the more people are infatuated with their beloved, the less they think they can up- and down-regulate infatuation, attachment, and sexual desire (hypothesis 5d) and that the more people are attached to their beloved, the less they think they can up- and down-regulate infatuation, attachment, and sexual desire (hypothesis 5e).

Finally, a meta-analysis has shown that some emotions are easier to regulate than others: down-regulation effects were larger for positive than negative affect, and for sadness than for fear and anger [46]. We already mentioned above that the perceived ability to regulate differs between different love feelings [3, 9]. Regardless of whether love is an emotion or not, it would be interesting to compare the perceived ability to up- and down-regulate different love types and emotions. Therefore, the sixth goal was to test whether people think they can up- and down-regulate infatuation, attachment, sexual desire, happiness, sadness, fear, and anger and whether people’s perceived ability to up- and down-regulate differs between infatuation, attachment, sexual desire, happiness, sadness, fear, and anger. Based on previous studies [3, 9, 46], we expected people to think they can up- and down-regulate happiness and sadness, but not fear, anger, infatuation, attachment, and sexual desire (hypothesis 6a). Also, we expected people to think that they can up- and down-regulate happiness and sadness most, and infatuation least (hypothesis 6b).

Please note that all six research questions concern whether people *think* they can regulate love. We used the self-report method to answer the research questions, because even though that method has its limitations, it is an important first step to assess *perceived* regulation ability [47].

## Materials and methods

### Participants

Unfortunately, there currently is no convenient way to perform a power analysis with more than one within-subject variable in G*Power (G*Power Feedback, personal communication, January 9, 2018). Instead, we decided to collect data until the end of the semester if we had useable data from at least 100 participants by that time. Three-hundred forty students of the University of Missouri-St. Louis volunteered to participate. Of those, 286 participants (mean age = 22.1 years, range = 18–39 years, 230 women, 52 men, 3 gender queers, 1 other) provided usable data (see below). Participants were recruited from the University of Missouri-St. Louis subject pool that includes students enrolled in Psychology courses. The study was advertised as Love Questionnaire Study. The study was approved by the Institutional Review Board of the University of Missouri-St. Louis (project number 919337). The consent form stated that the purpose of the study was to “understand how much control you feel you have over your emotions and love feelings”. Participants provided informed consent by clicking on an “agree’ button after reading the consent form on their computer screen. Participants were rewarded with course credit.

### Procedure

Participants completed a series of items online via Qualtrics and were allowed to skip items that they did not want to answer. Participants first completed demographic items including age, sex assigned at birth (male, female, intersex, other), what gender they currently identify with (man, women, gender queer, other), and their sexual orientation (straight, gay, bisexual, undecided, other). Additionally, participants reported whether they were currently in love or not. If so, they reported infatuation and attachment intensities on a 9-point Likert scale (1 = not at all, 9 = very much). Definitions of infatuation and attachment were provided at the top of each page of the online survey: “Infatuation (or passionate love) is the overwhelming amorous feeling for that one special person that is often accompanied by euphoria, butterflies in the stomach, and nervousness. This feeling does not have to be sexual.” and “Attachment (or companionate love) is a comforting feeling of emotional bonding with someone. This feeling does not have to be sexual.” We did not provide a definition for the word “love”, because it is very common in everyday language (as opposed to infatuation and attachment). Participants also reported whether they were currently in a romantic relationship or not. If so, they reported the relationship status (not married and not living together, not married and living together, married). If not, they reported whether they had ever been in a romantic relationship or not. Participants who were not currently in love reported whether they had ever been in love or not.

Then, participants completed several items that assessed perceived ability to up- and down-regulate love and emotions, exaggerate and suppress the expression of love, and start and stop love, see Table 1, in pseudorandom order. We varied the wording of the items (e.g., increase/decrease vs. enhance/reduce, I can/I can decide/I can choose) to reduce boredom in participants. We did make sure that items that were compared directly used the same words or the related antonym. These items were derived from a previous study [3], but were different in four ways. Firstly, the current items assessed the perceived ability to regulate different love types (i.e., infatuation, attachment, sexual desire) and emotions (i.e., happiness, sadness, fear, anger). These specific emotions were chosen because research has shown that they are considered the most prototypical emotions [13]. Secondly, besides infatuation and attachment, the current items assessed perceived ability to regulate sexual desire as well. Thirdly, the items also measured people’s perceived ability to exaggerate and suppress and start and stop love. Finally, the current items did not require participants to be in love.

**Table 1:** Mean scores on the items assessing perceived ability to regulate love and emotions.

Item	Construct	Statement	M	SD
1	Love-Up	I can increase my love.	6.4	2.1
2	Love-Down	I can decrease my love.	4.4	2.4
3	Infatuation-Up	I can enhance feelings of infatuation.	5.8	2.1
4	Attachment-Up	I can enhance feelings of attachment.	6.7	1.9
5	Sex-Up	I can enhance sexual desire.	6.3	2.0
6	Infatuation-Down	I can reduce feelings of infatuation.	4.8	2.1
7	Attachment-Down	I can reduce feelings of attachment.	4.8	2.3
8	Sex-Down	I can reduce sexual desire.	5.2	2.2
9	Infatuation-Exaggeration	I can choose to exaggerate expressions of infatuation.	6.5	2.1
10	Attachment-Exaggeration	I can choose to exaggerate expressions of attachment.	6.5	1.9
11	Sex-Exaggeration	I can choose to exaggerate expressions of sexual desire.	6.9	1.9
12	Infatuation-Suppression	I can decide to hide expressions of infatuation.	6.4	2.2
13	Attachment-Suppression	I can decide to hide expressions of attachment.	6.6	1.9
14	Sex-Suppression	I can decide to hide expressions of sexual desire.	6.7	2.1
15	Infatuation-Start	I can generate feelings of infatuation from scratch.	3.8	2.3
16	Attachment-Start	I can generate feelings of attachment from scratch.	4.1	2.4
17	Sex-Start	I can generate feelings of sexual desire from scratch.	4.5	2.6
18	Infatuation-Stop	I can decide to stop being infatuated with someone.	4.4	2.6
19	Attachment-Stop	I can decide to stop being attached to someone.	4.2	2.6
20	Sex-Stop	I can decide to stop feeling sexual desire for someone.	4.7	2.6
21	Happiness-Up	I can enhance feelings of happiness.	6.6	1.8
22	Sadness-Up	I can enhance feelings of fear.	6.3	2.0
23	Fear-Up	I can enhance feelings of fear.	5.2	2.2
24	Anger-Up	I can enhance feelings of anger.	6.3	2.0
25	Happiness-Down	I can reduce feelings of happiness.	6.6	1.8
26	Sadness-Down	I can reduce feelings of sadness.	6.3	2.3
27	Fear-Down	I can reduce feelings of fear.	5.1	2.2
28	Anger-Down	I can reduce feelings of anger.	5.4	2.3

*Note*. Items were presented to the participants in pseudorandom order (i.e., 1, 4, 18, 26, 11, 2, 3, 19, 23, 14, 7, 15, 21, 9, 8, 24, 6, 13, 17, 22, 10, 27, 5, 16, 25, 12, 28, 20). M = mean, SD = standard deviation. 1 = totally disagree, 5 = neutral, 9 = totally agree.

Each construct (e.g., perceived ability to down-regulate infatuation), see Table 1, was measured using a single item. Although it is necessary to use multiple items to measure complex constructs that cannot be assessed directly (such as personality), simpler constructs that can be assessed directly (such as job satisfaction, health-related quality of life, and even depression) can be measured adequately using a single item [48–50]. Likewise, perceived ability to regulate seems sufficiently narrow and unambiguous to be suitable for measurement with a single item. Participants indicated to what extent they agreed with each item on a 9-point Likert scale (1 = totally disagree, 5 = neutral, 9 = totally agree).

Additionally, three attention-checking items (e.g., Please select “strongly agree” to indicate that you have read this statement) were included to check whether participants actually read the statements before responding. Participants who did not answer all three attention-checking items correctly (*n* = 54) were excluded from the analysis, leaving the previously reported 286 participants. Finally, participants completed the Emotion Regulation Questionnaire (ERQ) [51] to assess the habitual use of cognitive reappraisal and expressive suppression. The ERQ consists of 10 items, six of which comprise the reappraisal subscale and four of which comprise the suppression subscale. An example of a Reappraisal item is “When I want to feel more *positive* emotion (such as joy or amusement), I *change what I’m thinking about*.” An example of a Suppression item is “When I am feeling *positive* emotions, I am careful not to express them.” The reappraisal and suppression subscales are the only two subscales of the ERQ.

### Analyses

There were 0.7% missing values on the items regarding perceived ability to regulate love and emotions. Participants with missing values were excluded from any analyses that involved the missed items(s) but were included in the other analyses. There were 0.4% missing values on the ERQ and these were replaced with the participant’s mean scores on the subscale. Mean scores on the reappraisal and suppression ERQ subscales were calculated [51].

To test whether people think that they cannot up- or down-regulate love in general (hypothesis 1a), one-sample t-tests against 5 (= neutral) were performed on the scores on items 1 and 2. To test whether people think that they can up-regulate love in general less than down-regulate love in general (hypothesis 1b), the scores on items 1 and 2 were compared with a paired samples t-test. To test whether people think that they cannot up- or down-regulate infatuation, attachment, and sexual desire, one-sample t-tests against 5 (= neutral) were performed on the scores on items 3–8 (hypothesis 2a). To test whether people think that they can up- and down-regulate infatuation least (hypothesis 2b), a repeated measure analysis of variance (rmANOVA) with the factors Direction (up-regulate, down-regulate) and Love Type (infatuation, attachment, sexual desire) was conducted on the scores on items 3–8. To test whether people think that they can exaggerate and suppress expressions of infatuation, attachment, and sexual desire (hypothesis 3), one sample t-tests against 5 (= neutral) were performed on the scores on items 9–14. To test whether people think that they cannot start and stop infatuation, attachment, and sexual desire (hypothesis 4), one sample t-tests against 5 (= neutral) were performed on the scores on items 15–20.

To test individual differences, various analyses were conducted. First, to test whether habitual use of cognitive reappraisal is positively associated with perceived ability to up- and down-regulate infatuation, attachment, and sexual desire (hypothesis 5a), an analysis of covariance (ANCOVA) with the continuous predictor ERQ Reappraisal (i.e., the mean score on the ERQ reappraisal subscale) and the factors Direction (up-regulate, down-regulate) and Love Type was conducted on the scores on items 3–8. Second, to test whether habitual use of expressive suppression is positively associated with perceived ability to suppress expressions of infatuation, attachment, and sexual desire (hypothesis 5b), an ANCOVA with the continuous predictor ERQ Suppression (i.e., the mean score on the ERQ suppression subscale), and the factors Direction (exaggerate, suppress) and Love Type was performed on the scores on items 9–14. Third, to test whether people who are in love think they can up- and down-regulate infatuation, attachment, and sexual desire less than people who are not in love (hypothesis 5c), a rmANOVA with the factors Being in Love (in love, not in love), Direction (up-regulate, down-regulate), and Love Type was conducted on the scores on items 3–8. Fourth, to test whether infatuation intensity is negatively associated with perceived ability to up- and down-regulate infatuation, attachment, and sexual desire (hypothesis 5d), an ANCOVA with the continuous predictor Infatuation Intensity (i.e., self-reported infatuation level) and the factors Direction (up-regulate, down-regulate) and Love Type was conducted on the scores on items 3–8. Finally, to test whether attachment intensity is negatively associated with perceived ability to up- and down-regulate infatuation, attachment, and sexual desire (hypothesis 5e), an ANCOVA with the continuous predictor Attachment Intensity (i.e., self-reported attachment level) and the factors Direction (up-regulate, down-regulate) and Love Type was conducted on the scores on items 3–8. See S1 Text for analyses concerning the effect of age on perceived ability to regulate. Please note that we are not using these ANCOVAs to control for individual differences, but that we are actually interested in the main and interaction effects that include the continuous predictors. Only effects involving the individual differences continuous predictors and factors are reported.

To test whether people think they can up- and down-regulate happiness and sadness, but not fear, anger, infatuation, attachment, and sexual desire (hypothesis 6a), one-sample t-tests against 5 (= neutral) were performed on the scores on items 3–8 and 21–28. To test whether people think that they can up- and down-regulate happiness and sadness most, and infatuation least (hypothesis 6b), a rmANOVA was performed with factors Direction (up-regulate, down-regulate) and Feeling (infatuation, attachment, sexual desire, happiness, sadness, anger, fear) on the scores on items 3–8 and 21–28.

When applicable, the degrees of freedom were corrected using the Greenhouse-Geisser correction. The *F* values, uncorrected degrees of freedom, the ε values, and corrected probability values are reported. A significance level of 5% (two-sided) was selected. Follow-up tests included paired samples t-tests for within-subject effects and independent samples t-tests for between-subject effects. To follow up significant effects of continuous predictors, Pearson correlation coefficients were computed. The Bonferroni correction was used for all follow-up tests to reduce the chance of Type I errors. The corrected α levels values are reported.

## Results

### Participant characteristics

Fifty-four participants (18.9%) were assigned male at birth and 232 participants (81.1%) were assigned female at birth. Moreover, 52 participants (18.2%) identified as men, 230 (80.4%) identified as women, 3 (1.4%) identified as gender queer, and 1 (0.3%) identified as other. In terms of sexual orientation, 233 participants (81.5%) reported being straight, 17 (5.9%) reported being gay, 27 (9.4%) reported being bisexual, 5 (1.7%) were undecided, and 2 (0.7%) reported being pansexual and/or queer.

One hundred eighty participants (62.9%) reported being in love, 84 participants (29.4%) reported to not be in love, but to have been in the past, 21 participants (7.3%) reported they had never been in love, and 1 participant (0.3%) did not report their love status. Participants who were in love reported an average infatuation intensity of 7.3 (SD = 1.7) and an average attachment intensity of 8.2 (SD = 1.2). Furthermore, 178 participants (62.2%) reported that they were currently in a romantic relationship, 84 participants (29.4%) reported not being in a relationship currently, but to have been in a romantic relationship in the past, 23 participants (8.0%) reported that they had never been in a romantic relationship, and 1 participant (0.3%) did not report their relationship status.

The mean score on the ERQ reappraisal subscale was 5.4 (SD = 1.1). The mean score on the ERQ suppression subscale was 3.4 (SD = 1.3).

### Perceived ability to regulate

See Table 1 for the mean scores on each item. See Table A in S1 Text for the correlations between items. Fig A in S1 Text for the distributions of the item scores.

#### Up- and down-regulation of love in general

Hypothesis 1a. We expected people to think that they cannot up- or down-regulate love in general. The mean score on the item assessing perceived ability to up-regulate love in general was significantly higher than 5 (= neutral), *t*(285) = 11.2, *p* < .001, whereas the mean score on the item assessing perceived ability to down-regulate love in general was significantly lower than 5 (= neutral), *t*(285) = -4.0, *p* < .001, Cohen’s *d* = .87. This indicates that participants thought that they could up- but not down-regulate love in general.

Hypothesis 1b. We expected people to think that they can up-regulate love in general less than down-regulate love in general. In contrast, participants thought that they could up-regulate love in general more than they could down-regulate love in general, *t*(285) = 12.2, *p* < .001, Cohen’s *d* = .87.

#### Up- and down-regulation of different love types

Hypothesis 2a. We expected people to think that they cannot up- or down-regulate infatuation, attachment, and sexual desire. The mean score on the items assessing perceived ability to up-regulate infatuation, attachment, and sexual desire were significantly higher than 5 (= neutral), all *ts*>6.7, all *ps* < .001, all Cohen’s *ds*>.40, whereas the mean score on the items assessing perceived ability to down-regulate infatuation, attachment, and sexual desire were not significantly different from 5 (= neutral), all *ts*<1.2, all *ps*>.13. This indicates that participants thought that they could up-regulate infatuation, attachment, and sexual desire, but that they felt neutral about their ability to up-regulate infatuation, attachment, and sexual desire.

**Fig 1:**
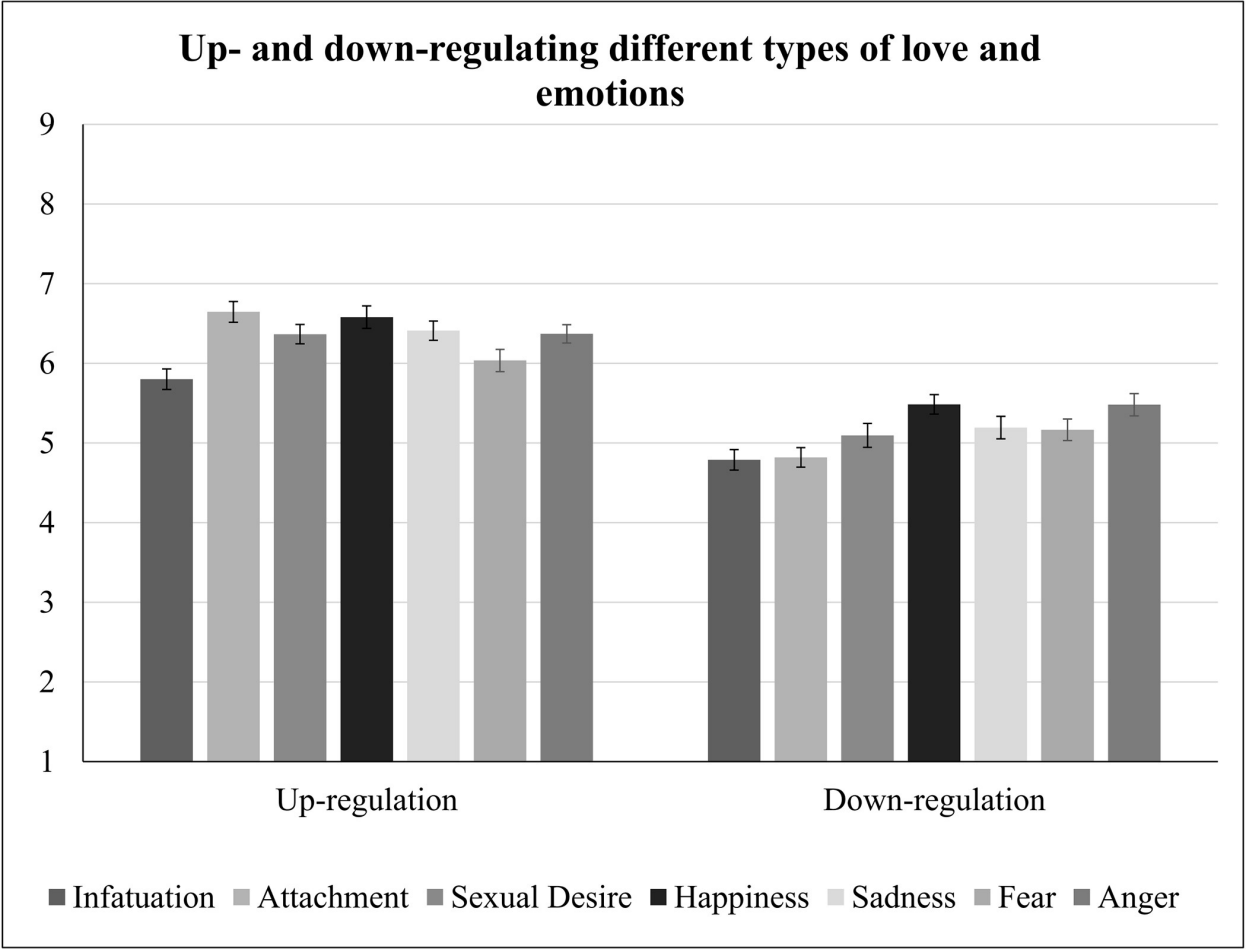
Perceived ability to up- and down-regulate infatuation, attachment, sexual desire, happiness, sadness, fear, and anger. Error bars indicate the standard error of the mean.

Hypothesis 2b. We expected people to think that they can up- and down-regulate infatuation least. There were main effects of Direction, *F*(1,270) = 133.5, *p* < .001, and Love Type, *F*(2,540) = 10.2, ε = .86 *p* < .001, but these were modulated by a Direction x Love Type interaction, *F*(2,540) = 13.0, ε = .86, *p* < .001, *η*^*2*^ = .046. Participants thought that they could up-regulate infatuation less than attachment and sexual desire, both *ps* < .001, all other *ps*>.016 (corrected α = .008), see Fig 1.

#### Exaggeration and suppression of different love types

Hypothesis 3. We expected people to think that they can exaggerate and suppress expressions of infatuation, attachment, and sexual desire. The mean scores on the items assessing perceived ability to exaggerate and suppress infatuation, attachment, and sexual desire were significantly higher than 5 (= neutral), all *ts*>10.7, all *ps* < .001, Cohen’s *ds*>.63. This indicates that participants thought that they could exaggerate and suppress expressions of infatuation, attachment, and sexual desire.

#### Starting and stopping different love types

Hypothesis 4. We expected people to think that they cannot start and stop infatuation, attachment, and sexual desire. The mean scores on the items assessing perceived ability to start and stop infatuation and attachment, and to start sexual desire were significantly lower than 5 (= neutral), all *ts*<-3.2, all *ps* ≤ .001, Cohen’s *d*s<-.20. The mean score on the item assessing perceived ability to stop sexual desire was not significantly different from 5 (= neutral), *t*(285) = -1.7, *p* = .10. This suggests that participants thought that they could not start and stop infatuation and attachment, and that they could not start sexual desire.

#### Individual differences

**Fig 2:**
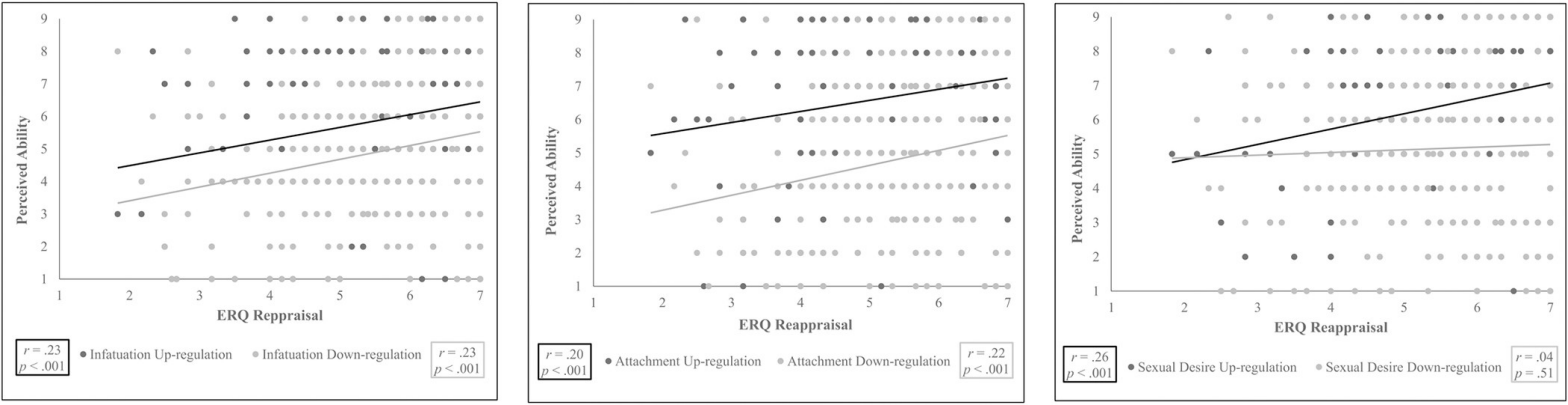
Correlations between the ERQ reappraisal score and perceived ability to up- and down-regulate different love types (corrected α = .008).

Hypothesis 5a. We expected that the more people habitually use cognitive reappraisal, the more they think they can up- and down-regulate infatuation, attachment, and sexual desire. There was an ERQ Reappraisal x Love Type x Direction interaction, *F*(2,538) = 5.6, ε = .87, *p =* .020, *η*^*2*^ = .030. The ERQ reappraisal score correlated positively with perceived ability to up-regulate infatuation, *r*(281) = .23, *p* < .001, attachment, *r*(283) = .20, *p =* .001, and sexual desire, *r*(282) = .26, *p* < .001, and perceived ability to down-regulate infatuation, *r*(280) = .23, *p* < .001, and attachment, *r*(283) = .22, *p <* .001, but not sexual desire, *r*(284) = .04, *p =* .51 (corrected α = .008), see Fig 2.

Hypothesis 5b. We expected that the more people habitually use expressive suppression, the more they think they can suppress expressions of infatuation, attachment, and sexual desire. There was an ERQ Suppression x Direction interaction, *F*(1,280) = 8.5, *p* = .004, *η*^*2*^ = .030, but neither of the follow up tests were significant, both *ps*>.041 (corrected α = .025).

Hypothesis 5c. We expected that people who are in love think they can up- and down-regulate infatuation, attachment, and sexual desire less than people who are not in love. There was a Being in Love x Direction interaction, *F*(1,269) = 6.1, ε = .87, *p =* .01, *η*^*2*^ = .022, but none of the follow-up tests were significant, all *ps*>.07 (corrected α = .025).

**Fig 3:**
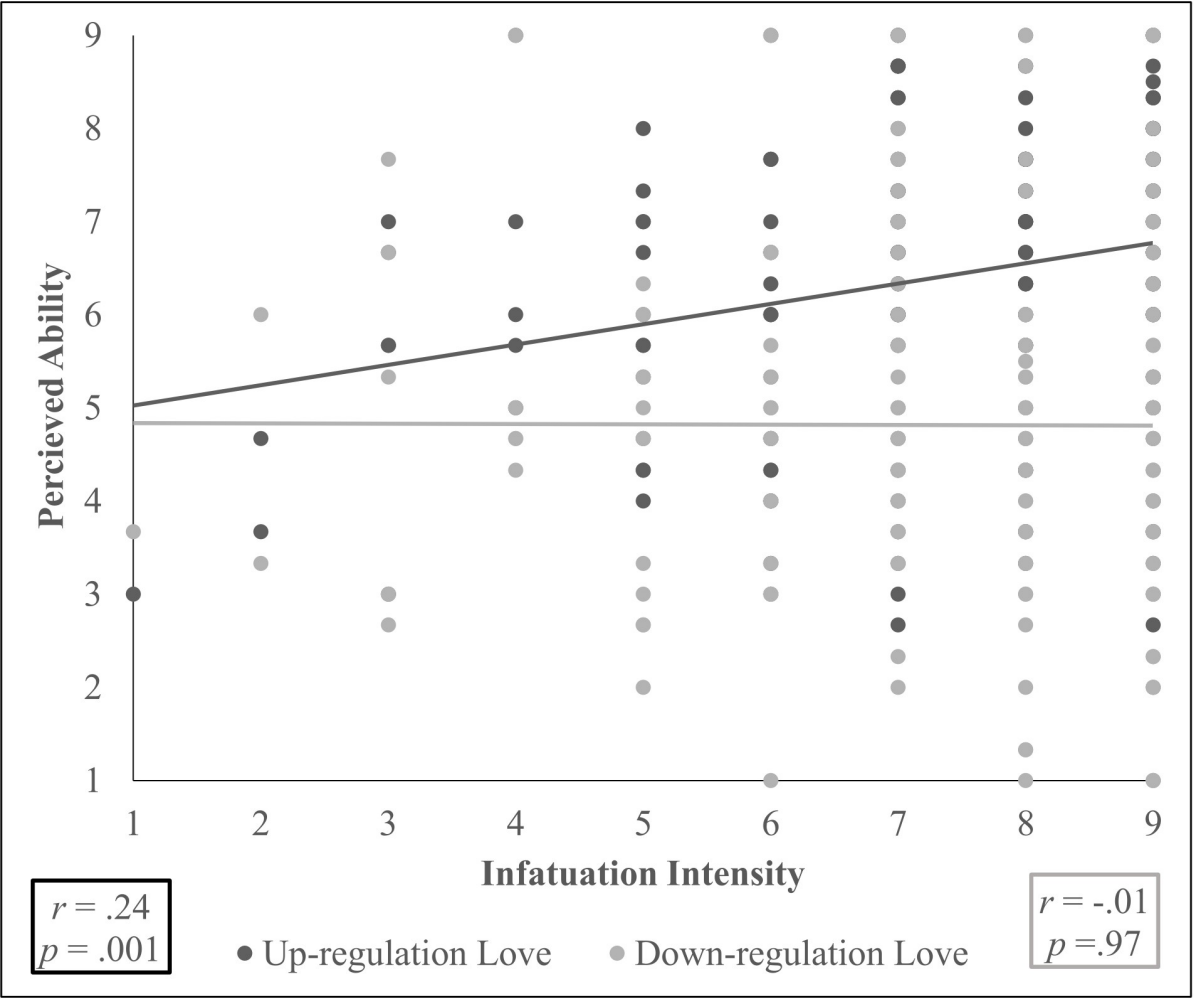
Correlations between self-reported infatuation intensity and perceived ability to up- and down-regulate love, collapsed across different love types (corrected α = .025).

Hypothesis 5d. We expected that the more people are infatuated with their beloved, the less they think they can up- and down-regulate infatuation, attachment, and sexual desire. There was an Infatuation Intensity x Direction interaction, *F*(1,168) = 6.8 , *p =* .01, *η*^*2*^ = .039. The more participants were infatuated with their beloved, the more they thought that they could up-regulate, *r*(180) = .24, *p =* .001, but not down-regulate, *r*(180) = -.01 *p =* .97 (corrected α = .025), love collapsed across the different love types, see Fig 3.

Hypothesis 5e. We expected that the more people are attached to their beloved, the less they think they can up- and down-regulate infatuation, attachment, and sexual desire. However, none of the effects involving Attachment Intensity were significant, all *Fs*<6.7, all *ps*>.62.

#### Up- and down-regulation of love types and emotions

Hypothesis 6a. We expected people to think they can up- and down-regulate happiness and sadness, but not fear, anger, infatuation, attachment, and sexual desire (hypothesis 6a). The mean score on the items assessing perceived ability to up-regulate infatuation, attachment, sexual desire, happiness, sadness, fear, and anger were significantly higher than 5 (= neutral), all *ts*>6.7, all *ps* < .001, all Cohen’s *ds*>.20. The mean score on the items assessing perceived ability to down-regulate happiness and anger were significantly higher than 5 (= neutral), both *ts*>3.3, both *ps* ≤ .001, Cohen’s *ds*>.46, and the mean scores on items assessing perceived ability to down-regulate infatuation, attachment, sexual desire, sadness, and fear were not significantly different from 5 (= neutral), all *ts*<1.3, all *ps*>.10. This indicates that participants thought that they could up-regulate infatuation, attachment, sexual desire, happiness, sadness, fear, and anger, and down-regulate happiness and anger.

Hypothesis 6b. We expected people to think that they can up- and down-regulate happiness and sadness most, and infatuation least. There were main effects of Direction, *F*(1,253) = 179.8, *p* < .001, *η*^*2*^ = .42, and Feeling, *F*(6,1518) = 9.2, ε = .89, *p* < .001, *η*^*2*^ = .035, but these were modulated by a Direction x Feeling interaction, *F*(6,1518) = 4.2, ε = .82, *p =* .001, *η*^*2*^ = .016, see Fig 1. Participants thought that they could up-regulate attachment and happiness more than infatuation and fear, all *p*s < .001. Participants also thought that they could up-regulate sexual desire more than infatuation, *p* < .001. Finally, participants thought that they could down-regulate happiness and anger more than infatuation and attachment, all *p*s < .001 (corrected α = .001).

## Discussion

Previous studies have shown that love can be up- and down-regulated [3–9]. The current study investigated people’s perceptions about love regulation because those may impact mental health and may influence whether people will actually engage in love regulation. To this end, participants completed items that assessed the extent to which they think they could up- and down-regulate love, exaggerate and suppress the expression of love, and start and stop love. We also tested individual differences in perceptions regarding love regulation, and differences in perceptions between infatuation, attachment, sexual desire, happiness, sadness, fear, and anger.

The first goal of the study was to investigate was to investigate whether people think they can up- and down-regulate love in general and whether the perceived ability to regulate love in general differs between up- and down-regulation. We expected people to think that they cannot up- or down-regulate love in general (hypothesis 1a) and to think that they can up-regulate love less than down-regulate love in general (hypothesis 1b). Participants thought that they could up- but not down-regulate love in general, which partially confirms hypothesis 1a. In previous studies, participants thought that love in general is somewhat uncontrollable and that down-regulation of love in general is neither possible nor impossible [3, 9, 37]. Because previous studies have shown that love up- and down-regulation are actually feasible when people are given explicit instructions on how to regulate their love feelings [3–9], the perception that one cannot (down-)regulate love is incorrect. Additionally, participants thought that they could up-regulate love in general more than they could down-regulate love in general, which was opposite to hypothesis 1b. In a previous study, participants seemed more successful at down- than up-regulating infatuation and attachment [3]. So again, there appears to be a mismatch between perceptions of ability to regulate love and actual regulation ability. It should be noted, however, that we did not assess actual regulation ability in the current sample. So, it may be that people are unable to regulate love until they received explicit instructions on how to regulate it, which would correspond with their perceptions. So, future studies could assess perceived ability to regulate love after participants performed a regulation task with explicit instructions on how to regulate love to more definitely conclude whether perceived and actual regulation ability match or not.

The second goal was to test whether people think they can up- and down-regulate infatuation, attachment, and sexual desire, and whether people’s perceived ability to up- and down-regulate differs between infatuation, attachment, and sexual desire. We expected people to think that they cannot up- or down-regulate infatuation, attachment, and sexual desire either (hypothesis 2a). Participants thought they could up-regulate infatuation, attachment, and sexual desire and felt neutral about their ability to down-regulate infatuation, attachment, and sexual desire, which is in contrast to hypothesis 2a. However, it is in line with previous findings that participants thought that down-regulation of love in general was neither possible nor impossible [9]. We also expected people to think that they can up- and down-regulate infatuation least (hypothesis 2b). Participants thought that they could up-regulate infatuation less than attachment and sexual desire, which aligns with hypothesis 2b. This also corresponds with the previous finding that people perceived less control over non-sexual than sexual intrusive thoughts [4], that people felt less in control of feelings of infatuation than attachment [3], and that positive reappraisal increased attachment, but not infatuation, in a love regulation task [3]. In contrast to hypothesis 2, no differences were observed between infatuation, attachment, and sexual desire in the perceived ability to down-regulate. Nevertheless, this is in line with previous findings that perceived ability to down-regulate did not differ between infatuation and attachment [9], and that negative reappraisal decreased both infatuation and attachment in a love regulation task [3]. The current study extends the previous work by showing that perceived regulation feasibility varies between different love types and regulation directions. Future studies could compare the actual feasibility of up- and down-regulation of the three love types infatuation, attachment, and sexual desire.

The third goal was to investigate whether people think they can exaggerate and suppress expressions of infatuation, attachment, and sexual desire. We expected people to think that they can exaggerate and suppress expressions of infatuation, attachment, and sexual desire (hypothesis 3). Participants thought that they could exaggerate and suppress expressions of infatuation, attachment, and sexual desire, which aligns with hypothesis 3. Previous studies have shown that emotional expressions can be exaggerated and suppressed [38, 39] and that love is expressed through head nods, Duchenne smiles, gesticulation, forward leans, blushing, and pupil dilation [14, 40]. Therefore, future studies could examine whether expressions of infatuation, attachment, and sexual desire can be exaggerated and suppressed as well.

The fourth goal was to investigate whether people think they can start and stop infatuation, attachment, and sexual desire. We expected people to think that they cannot start and stop infatuation, attachment, and sexual desire (hypothesis 4). Participants thought that they could not start infatuation, attachment, and sexual desire, or stop infatuation and attachment. This mostly aligns with hypothesis 4 and corresponds with the finding from the previous study that showed that participants felt more in control of the intensity of love than the object of love [3]. It is worth noting that participants may have interpreted the items about starting love as generating love for someone they just met or as generating love for a person they already knew. The current items do not distinguish between these two types of starting love feelings. It would be interesting to test if perceived ability to start love depends on whether it involves generating love for a person you already know vs. generating love for a person you have just met. Because people can initiate new emotions [42, 43], it would also be interesting to investigate whether people can actually start and stop love.

The fifth goal was to investigate individual differences in the perceived ability to up- and down-regulate infatuation, attachment, and sexual desire. First, we tested the effect of habitual use of cognitive reappraisal. We expected that the more people habitually use cognitive reappraisal, the more they think they can up- and down-regulate infatuation, attachment, and sexual desire (hypothesis 5a). The ERQ reappraisal score was positively correlated with perceived ability to up- and down-regulate infatuation and attachment and to up-regulate sexual desire, which was mostly in line with hypothesis 5a. This means that the more people habitually use cognitive reappraisal to regulate emotions, the more they think that they can up- and down-regulate infatuation and attachment and up-regulate sexual desire. This finding is in line with the previous finding that perceived control over infatuation and attachment was positively associated with habitual use of reappraisal [3] and extends that previous finding by showing that the habitual use of cognitive reappraisal is positively associated with perceived ability to both up- and down-regulate infatuation and attachment, as well as the perceived ability to up-regulate sexual desire. These perceptions about love regulation are also in line with the findings that cognitive reappraisal is an effective strategy to up-regulate attachment and down-regulate infatuation and attachment [3, 9]. Interestingly, there was no association between the habitual use of cognitive reappraisal for emotion regulation and the perceived ability to down-regulate sexual desire. This suggests that perhaps other regulation strategies may be more effective for down-regulating sexual desire than cognitive reappraisal. Although it has been shown that men who were able to regulate emotions such as amusement and humor were also able to effectively down-regulate sexual arousal using reappraisal [52], it has also been shown that individuals more often use behavioral distraction than cognitive reappraisal when they want to decrease intrusive sexual thoughts [4]. It would be interesting to test whether the habitual use of reappraisal for emotion regulation is related to actual love regulation ability.

Second, we tested the effect of habitual use of expressive suppression. We expected that the more people habitually use expressive suppression, the more they think they can suppress expressions of infatuation, attachment, and sexual desire (hypothesis 5b). However, the ERQ suppression score did not correlate significantly with the perceived ability to suppress, or exaggerate, love. Because the expression of feelings may be a continuum ranging from suppression to natural expression to exaggeration, it would be interesting to test whether the habitual use of suppression is related to actual ability to both exaggerate and suppress love expressions such as head nods, Duchenne smiles, gesticulation, forward leans, blushing, and pupil dilation [14, 40].

Third, we tested the effects of being in love, infatuation intensity, and attachment intensity. We expected that people who are in love think they can up- and down-regulate infatuation, attachment, and sexual desire less than people who are not in love (hypothesis 5c) and that the more people are infatuated with their beloved, the less they think they can up- and down-regulate infatuation, attachment, and sexual desire (hypothesis 5d). The perceived ability to regulate love did not differ between people who were and who were not in love, which was in contrast to hypothesis 5c. This means that being in love was not associated with participants’ perceived ability to regulate love. Nevertheless, there was a positive correlation between the self-reported infatuation level and perceived ability to up-regulate love, which was opposite to hypothesis 5d. This finding suggests that the more participants were infatuated, the more they thought that they could up- but not down-regulate love. We expected that the more people are attached to their beloved, the less they think they can up- and down-regulate infatuation, attachment, and sexual desire (hypothesis 5e). However, there was no correlation between the self-reported attachment level and perceived love regulation ability, which was not in line with hypothesis 5e. These findings are surprising because love regulation will likely require cognitive control, and because love intensity as measured by the Passionate Love Scale correlated negatively with cognitive control [45]. Future research could test whether being in love, and infatuation and attachment intensity are associated with the actual ability to up- and down-regulate infatuation, attachment, and sexual desire. Finally, future studies could test the perceived ability to regulate love in individuals that are in love but not in a relationship and in individuals that are in a relationship but not in love.

The sixth goal of the study was to test whether people think they can up- and down-regulate infatuation, attachment, sexual desire, happiness, sadness, fear, and anger and whether people’s perceived ability to up- and down-regulate differs between infatuation, attachment, sexual desire, happiness, sadness, fear, and anger. We expected people to think they can up- and down-regulate happiness and sadness, but not fear, anger, infatuation, attachment, and sexual desire (hypothesis 6a). People thought that they could up-regulate infatuation, attachment, sexual desire, happiness, sadness, fear, and anger, and down-regulate happiness and anger. These findings are partially in line with our hypothesis 6a. We also expected people to think that they can up- and down-regulate happiness and sadness most, and infatuation least (hypothesis 6b). People also thought that they could up-regulate attachment and happiness more than infatuation and fear. Plus, people thought that they could up-regulate sexual desire more than infatuation. Finally, people thought that they could down-regulate happiness and anger more than infatuation and attachment. These findings are mostly in line with hypothesis 6b, except that we did not observe the hypothesized effect for sadness. The observation that people think they can regulate happiness particularly well is in line with a meta-analysis that has shown that down-regulation effects were larger for positive than negative affect [46]. Although infatuation is associated with positive affect, it is also is associated with negative affect such as anxiety and nervousness [37, 53, 54], which could have contributed to the observed differences between happiness and infatuation. Future research could test whether actual ability to up- and down-regulate differs between infatuation, attachment, sexual desire, happiness, sadness, fear, and anger.

This study has some limitations. First, men were underrepresented in the sample, which likely results from the gender ratio of the population from which participants were sampled and perhaps also from the greater willingness of women to participate in studies about love. Second, the sample consisted of young adults who were college students. It should be noted that the institution that the data were collected at has a diverse student body and that the sample was diverse in terms of sexual orientation, current love status, and current relationship status. Additionally, our sample was much larger (*n* = 286) than the samples of previous studies about love regulation perceptions (*n* ≤ 40) [3, 9], which greatly increases the generalizability of the current findings. Third, it is worth noting that laypeople may not consider sexual desire a type of love, especially if it is not directed at a person who they also experience other types of love feelings (such as infatuation and/or attachment) for. However, responses to the items about sexual desire likely did not depend on whether participants viewed sexual desire as a type of love or not, because these items did not mention the word “love”. Fourth, each construct was measured using a single item. Although research has shown that simple constructs that can be assessed directly (such as job satisfaction, health-related quality of life, and depression, and presumably perceived ability to regulate) can be measured adequately using a single item [48–50], it might be good to confirm the current results using multiple items per construct. Finally, the individual differences were tested cross-sectionally and are hence only correlational. To establish causation, it could be tested whether changes in habitual use of reappraisal and expressive suppression (e.g., through training), falling in and out of love, and changes in infatuation and attachment intensity over time, change people’s perceived ability to regulate love.

To conclude, this study was the first to thoroughly examine perceptions about love regulation and individual differences in these perceptions in a large sample. Despite the fact that love can be up- and down-regulated [3–9], people think that they are not able carry out some forms of love regulation. These findings are a first step toward the development of psychoeducation techniques to correct inaccurate perceptions about love regulation. Because not feeling in control negatively affects mental and physical health [30, 31, 55, 56], learning that love can be regulated at least to some extent may be beneficial for mental and physical well-being. Additionally, because not feeling in control reduces the likelihood and effectiveness of regulation efforts [32–35], learning that love can be regulated may increase the likelihood and effectiveness of love regulation in daily life. These predictions await testing in future studies. Future studies could also investigate the perceived ability to regulate other types of love, such as compassionate and maternal love [21, 28], as well as cultural differences in perceived ability to regulate love. Love regulation could help people cope with break-ups and might increase the stability of long-term relationships.

## Supporting information

S1 Text Correlations between items, item distributions, and effect of age. (DOCX)
