# Supplementary material for: Perceived ability to regulate love
Source: PLoS One. 2019 May 13;14(5):e0216523. doi: 10.1371/journal.pone.0216523 (PMC6513268; doi:10.1371/journal.pone.0216523)
Supplement: S1 Text — (DOCX) [file pone.0216523.s001.docx]

**S1 Supporting Information**

**Correlations Between Items**

See Table A for the correlations between items. As can been seen, most items correlated positively with each other, which is probably due to the fact that they all tap into whether people think they can regulate their feelings. However, most correlations are small to moderate rather than large or perfect, which suggests that the items do tap into separate constructs as well.

In addition, as can be seen, perceived ability to up-regulate love in general was positively correlated with perceived ability to up-regulate infatuation, attachment, and sexual desire. Likewise, perceived ability to down-regulate love in general was positively correlated with perceived ability to down-regulate infatuation, attachment, and sexual desire. However, the correlations seem stronger between the items assessing perceived ability to up- and down-regulate love in general, and attachment and infatuation, than between the items assessing perceived ability to up- and down-regulate love in general and sexual desire. This suggests that people consider sexual desire to be less prototypical of love in general than attachment and infatuation.

| **Item** | **1** | **2** | **3** | **4** | **5** | **6** | **7** | **8** | **9** | **10** | **11** | **12** | **13** | **14** | **15** | **16** | **17** | **18** | **19** | **20** | **21** | **22** | **23** | **24** | **25** | **26** | **27** | **28** |
| --- | --- | --- | --- | --- | --- | --- | --- | --- | --- | --- | --- | --- | --- | --- | --- | --- | --- | --- | --- | --- | --- | --- | --- | --- | --- | --- | --- | --- |
| **1 Love Up** | 1 |  |  |  |  |  |  |  |  |  |  |  |  |  |  |  |  |  |  |  |  |  |  |  |  |  |  |  |
| **2 Love Down** | .28^**^ | 1 |  |  |  |  |  |  |  |  |  |  |  |  |  |  |  |  |  |  |  |  |  |  |  |  |  |  |
| **3 Infatuation Up** | .60^**^ | .35^**^ | 1 |  |  |  |  |  |  |  |  |  |  |  |  |  |  |  |  |  |  |  |  |  |  |  |  |  |
| **4 Attachment Up** | .75^**^ | .28^**^ | .59^**^ | 1 |  |  |  |  |  |  |  |  |  |  |  |  |  |  |  |  |  |  |  |  |  |  |  |  |
| **5 Sex Up** | .24^**^ | .11 | .30^**^ | .19^**^ | 1 |  |  |  |  |  |  |  |  |  |  |  |  |  |  |  |  |  |  |  |  |  |  |  |
| **6 Infatuation Down** | .28^**^ | .57^**^ | .30^**^ | .29^**^ | .22^**^ | 1 |  |  |  |  |  |  |  |  |  |  |  |  |  |  |  |  |  |  |  |  |  |  |
| **7 Attachment Down** | .18^**^ | .51^**^ | .15^*^ | .25^**^ | .12^*^ | .67^**^ | 1 |  |  |  |  |  |  |  |  |  |  |  |  |  |  |  |  |  |  |  |  |  |
| **8 Sex Down** | .09 | .16^**^ | -.02 | .07 | .20^**^ | .37^**^ | .27^**^ | 1 |  |  |  |  |  |  |  |  |  |  |  |  |  |  |  |  |  |  |  |  |
| **9 Infatuation Exaggeration** | .15^**^ | .11 | .28^**^ | .16^**^ | .25^**^ | .25^**^ | .14^*^ | .16^**^ | 1 |  |  |  |  |  |  |  |  |  |  |  |  |  |  |  |  |  |  |  |
| **10 Attachment Exaggeration** | .06 | .05 | .20^**^ | .08 | .25^**^ | .19^**^ | .15^*^ | .11 | .60^**^ | 1 |  |  |  |  |  |  |  |  |  |  |  |  |  |  |  |  |  |  |
| **11 Sex Exaggeration** | .13^*^ | -.02 | .16^**^ | .09 | .30^**^ | .14^*^ | .06 | .11 | .55^**^ | .50^**^ | 1 |  |  |  |  |  |  |  |  |  |  |  |  |  |  |  |  |  |
| **12 Infatuation Suppression** | .03 | .15^*^ | .06 | -.01 | .20^**^ | .31^**^ | .29^**^ | .23^**^ | .41^**^ | .45^**^ | .27^**^ | 1 |  |  |  |  |  |  |  |  |  |  |  |  |  |  |  |  |
| **13 Attachment Suppression** | .00 | .14^*^ | -.01 | -.01 | .16^**^ | .33^**^ | .33^**^ | .08 | .26^**^ | .37^**^ | .21^**^ | .53^**^ | 1 |  |  |  |  |  |  |  |  |  |  |  |  |  |  |  |
| **14 Sex Suppression** | -.09 | .09 | .03 | -.04 | .19^**^ | .27^**^ | .25^**^ | .25^**^ | .28^**^ | .24^**^ | .18^**^ | .41^**^ | .28^**^ | 1 |  |  |  |  |  |  |  |  |  |  |  |  |  |  |
| **15 Infatuation Start** | .35^**^ | .19^**^ | .34^**^ | .28^**^ | .24^**^ | .24^**^ | .12^*^ | .27^**^ | .11 | .07 | .05 | .01 | .04 | .01 | 1 |  |  |  |  |  |  |  |  |  |  |  |  |  |
| **16 Attachment Start** | .30^**^ | .19^**^ | .35^**^ | .29^**^ | .31^**^ | .27^**^ | .16^**^ | .23^**^ | .15^*^ | .12^*^ | .07 | .08 | .03 | .08 | .67^**^ | 1 |  |  |  |  |  |  |  |  |  |  |  |  |
| **17 Sex Start** | .27^**^ | .12^*^ | .27^**^ | .20^**^ | .43^**^ | .24^**^ | .08 | .16^**^ | .12^*^ | .11 | .12^*^ | .09 | .09 | .12^*^ | .56^**^ | .59^**^ | 1 |  |  |  |  |  |  |  |  |  |  |  |
| **18 Infatuation Stop** | .26^**^ | .52^**^ | .25^**^ | .26^**^ | .23^**^ | .56^**^ | .43^**^ | .21^**^ | .10 | .07 | .09 | .12 | .17^**^ | .09 | .20^**^ | .12^*^ | .18^**^ | 1 |  |  |  |  |  |  |  |  |  |  |
| **19 Attachment Stop** | .28^**^ | .55^**^ | .26^**^ | .28^**^ | .15^**^ | .56^**^ | .64^**^ | .22^**^ | .17^**^ | .09 | .05 | .20^**^ | .22^**^ | .14^*^ | .21^**^ | .22^**^ | .18^**^ | .57^**^ | 1 |  |  |  |  |  |  |  |  |  |
| **20 Sex Stop** | .27^**^ | .34^**^ | .21^**^ | .23^**^ | .18^**^ | .48^**^ | .38^**^ | .45^**^ | .15^*^ | .11 | .05 | .14^*^ | .15^*^ | .10 | .34^**^ | .31^**^ | .19^**^ | .40^**^ | .45^**^ | 1 |  |  |  |  |  |  |  |  |
| **21 Happy Up** | .28^**^ | .17^**^ | .37^**^ | .32^**^ | .21^**^ | .28^**^ | .23^**^ | .08 | .27^**^ | .13^*^ | -.04 | .15^*^ | .17 | .08 | .23^**^ | .27^**^ | .16^**^ | .22^**^ | .27^**^ | .20^**^ | 1 |  |  |  |  |  |  |  |
| **22 Sad Up** | .10 | .12^*^ | .16^**^ | .10 | .18^**^ | .21^**^ | .08 | .12^*^ | .31^**^ | .36^**^ | .26^**^ | .38^**^ | .21^**^ | .20^**^ | .08 | .13^*^ | .11 | .08 | .05 | .04 | .15^*^ | 1 |  |  |  |  |  |  |
| **23 Fear Up** | .17^**^ | .22^**^ | .27^**^ | .16^**^ | .13^*^ | .23^**^ | .15^*^ | .07 | .25^**^ | .16^**^ | .10 | .26^**^ | .09 | .14^*^ | .09 | .16^**^ | .04 | .08 | .17^**^ | .06 | .25^**^ | .40^**^ | 1 |  |  |  |  |  |
| **24 Anger Up** | .06 | .18^**^ | .20^**^ | .00 | .29^**^ | .27^**^ | .13^*^ | .21^**^ | .29^**^ | .31^**^ | .19^**^ | .35^**^ | .22^**^ | .18^**^ | .01 | .15^**^ | .13^*^ | .14^*^ | .16^**^ | .09 | .23^**^ | .49^**^ | .36^**^ | 1 |  |  |  |  |
| **25 Happy Down** | .08 | .19^**^ | .07 | .04 | .21^**^ | .21^**^ | .21^**^ | .23^**^ | .23^**^ | .21^**^ | .17^**^ | .38^**^ | .16^**^ | .09 | .11 | .18^**^ | .10 | .04 | .09 | .15^*^ | .18^**^ | .32^**^ | .32^**^ | .28^**^ | 1 |  |  |  |
| **26 Sad Down** | .19^**^ | .35^**^ | .25^**^ | .21^**^ | .20^**^ | .36^**^ | .39^**^ | .04 | .17^**^ | .05 | .07 | .21^**^ | .16^**^ | .09 | .07 | .14^*^ | .10 | .40^**^ | .49^**^ | .16^**^ | .38^**^ | .04 | .12^*^ | .07 | .14^*^ | 1 |  |  |
| **27 Fear Down** | .12^*^ | .24^**^ | .15^*^ | .16^**^ | .23^**^ | .39^**^ | .40^**^ | .27^**^ | .21^**^ | .22^**^ | .12 | .22^**^ | .25^**^ | .16^**^ | .16^**^ | .21^**^ | .20^**^ | .18^**^ | .39^**^ | .26^**^ | .16^**^ | .17^**^ | .17^**^ | .26^**^ | .26^**^ | .39^**^ | 1 |  |
| **28 Anger Down** | .18^**^ | .16^**^ | .17^**^ | .18^**^ | .28^**^ | .35^**^ | .31^**^ | .26^**^ | .18^**^ | .17^**^ | .09 | .23^**^ | .18^**^ | .09 | .18^**^ | .19^**^ | .16^**^ | .21^**^ | .34^**^ | .23^**^ | .31^**^ | .19^**^ | .20^**^ | .22^**^ | .32^**^ | .36^**^ | .56^**^ | 1 |
| ** Correlation is significant at the 0.01 level (2-tailed). | | | | | | | | | | | | | | | | | | | | | | | | | | | | |
| * Correlation is significant at the 0.05 level (2-tailed). | | | | | | | | | | | | | | | | | | | | | | | | | | | | |

**Table A. Correlations between the items assessing perceived ability to regulate love and emotions.**

**Item Distributions**

Even though we assessed the effect of several individual difference variables on perceived ability to regulate love, it might be that some variable(s) that we did not assess affected perceived ability to regulate love leading to a dichotomous distribution of some people who believe they are able to regulate love and others who believe they are not able to. Therefore, we assessed whether the distributions of the scores on the perceived feasibility items were distributed dichotomously into high and low scores. See Figure A for the histograms showing the distributions of all items. As can be seen, none of the items had a bimodal distribution.

**Fig. A Histograms of distributions of items measuring perceived ability to regulate.**


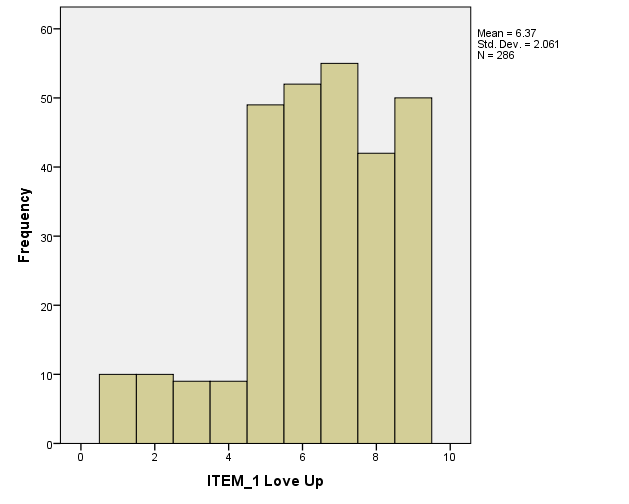

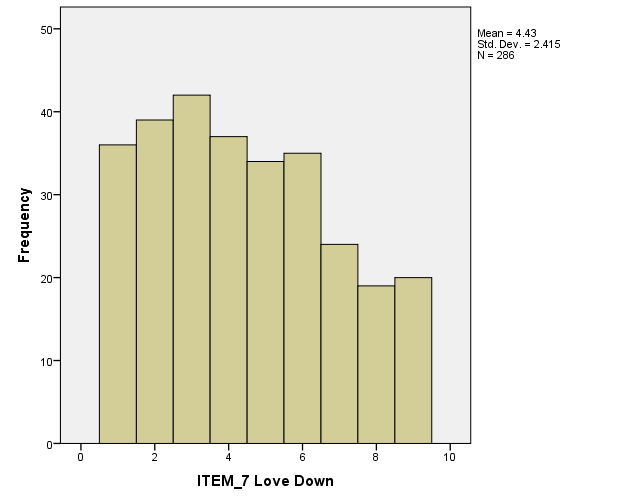


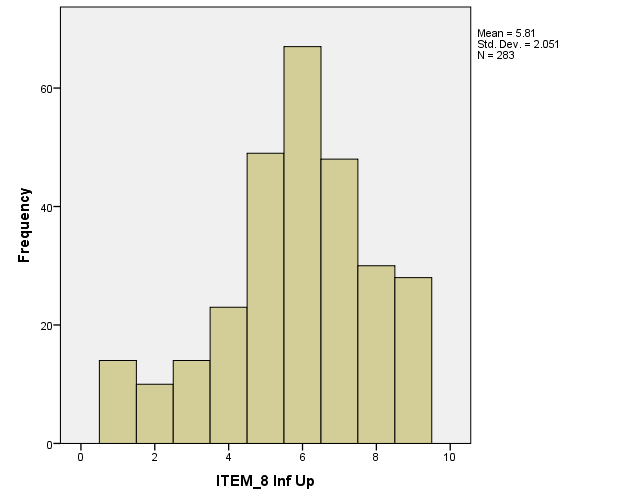

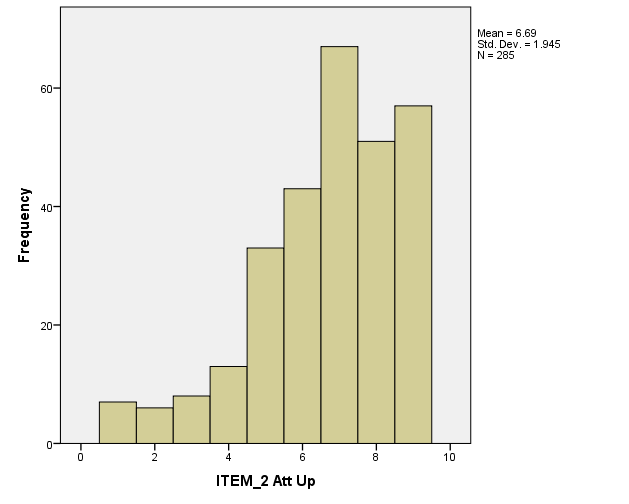


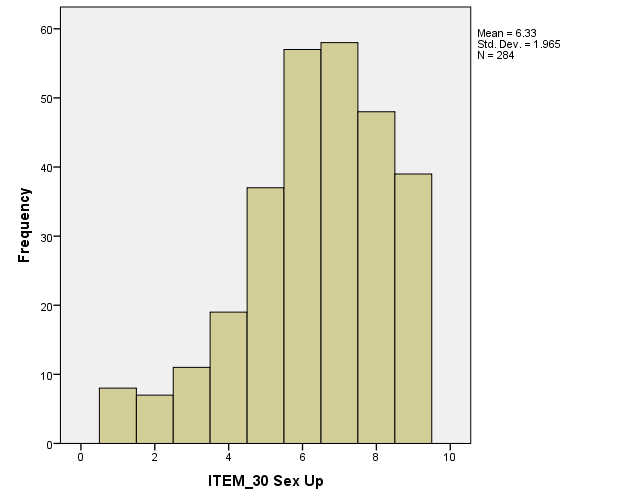

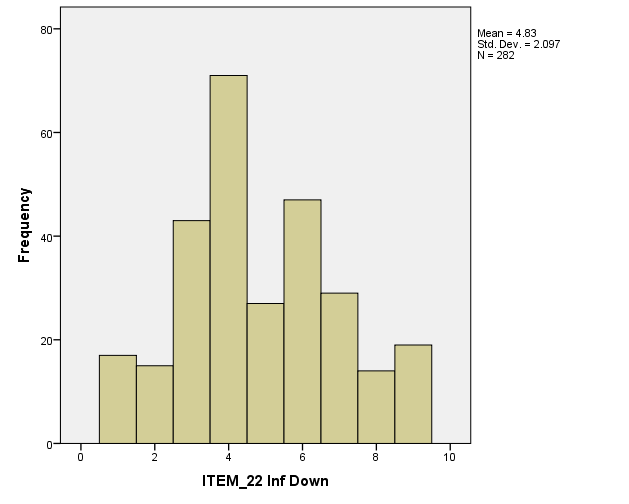


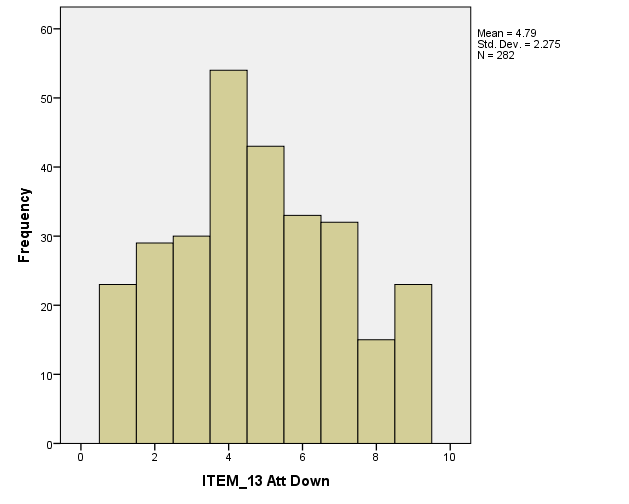

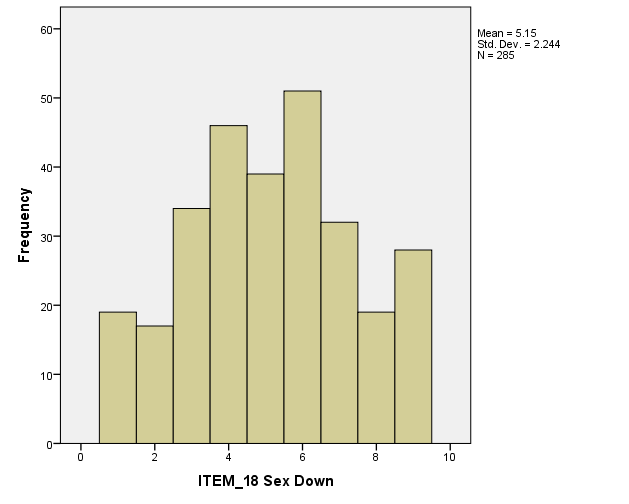


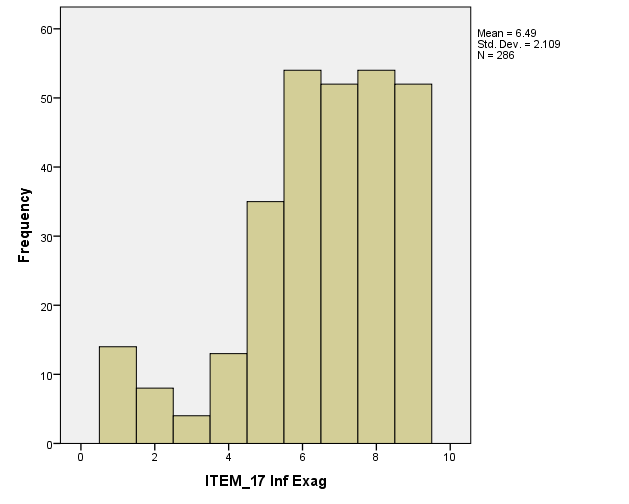

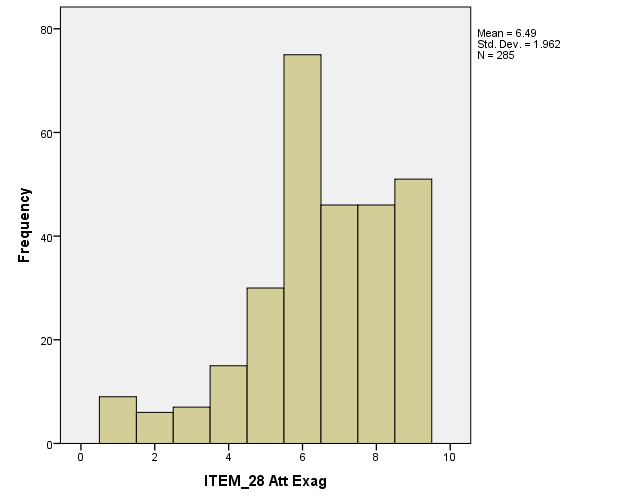


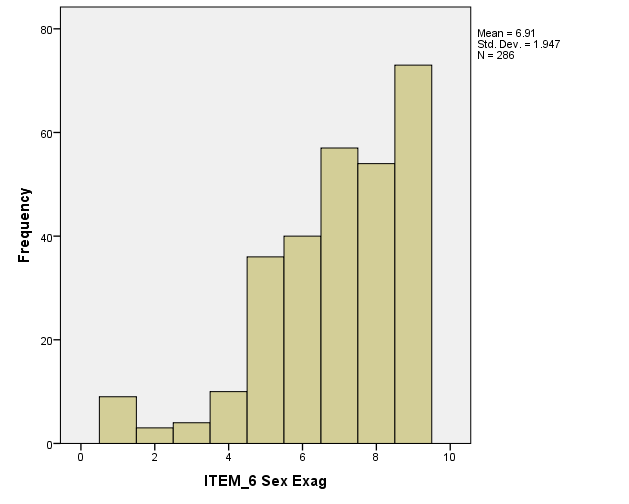

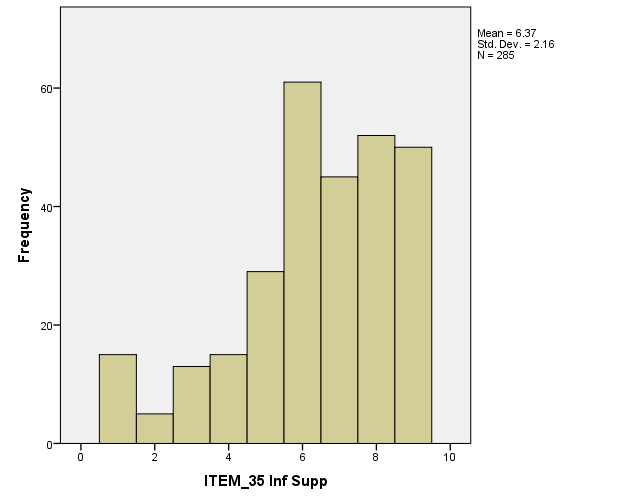


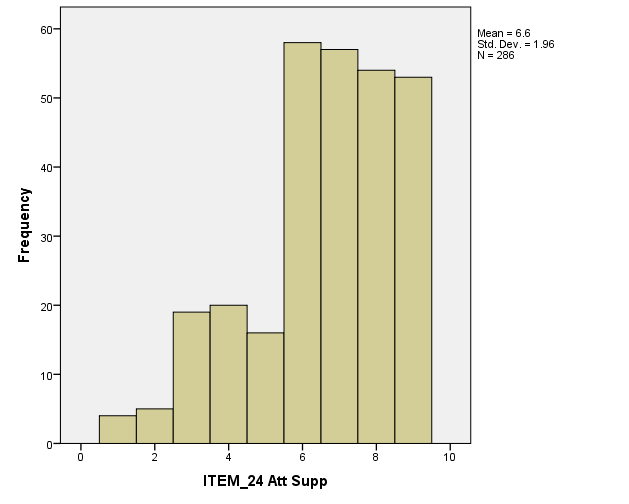

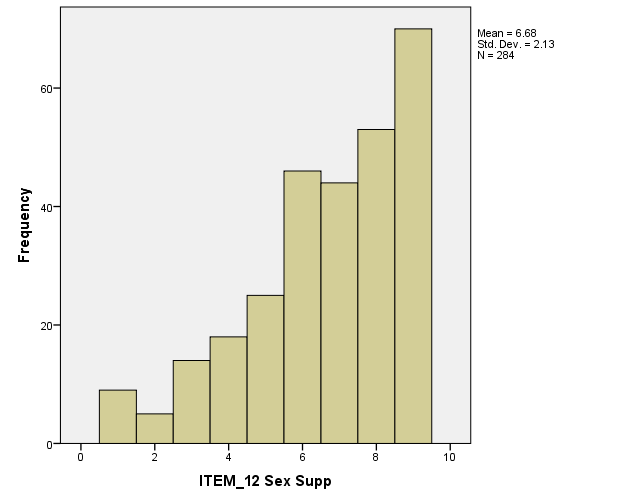


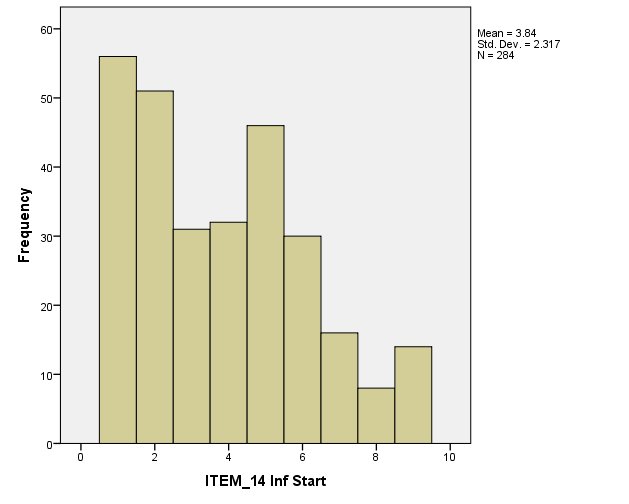

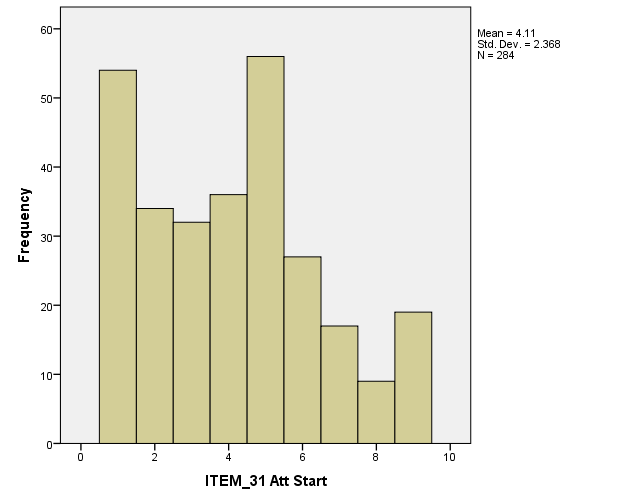


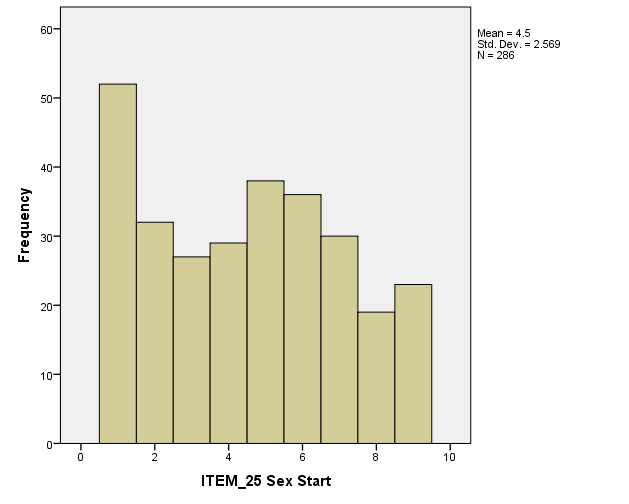

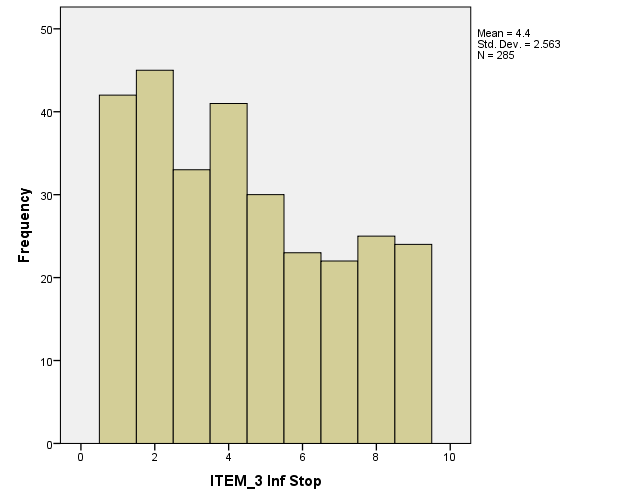


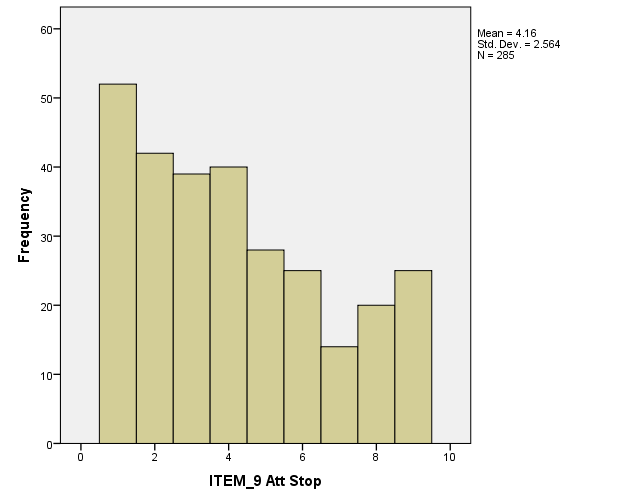

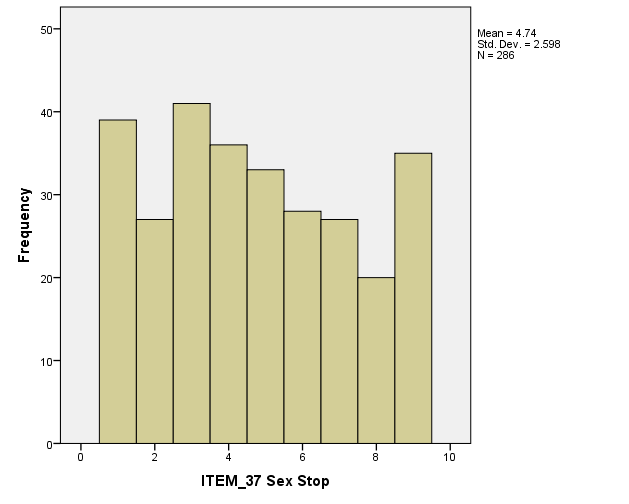


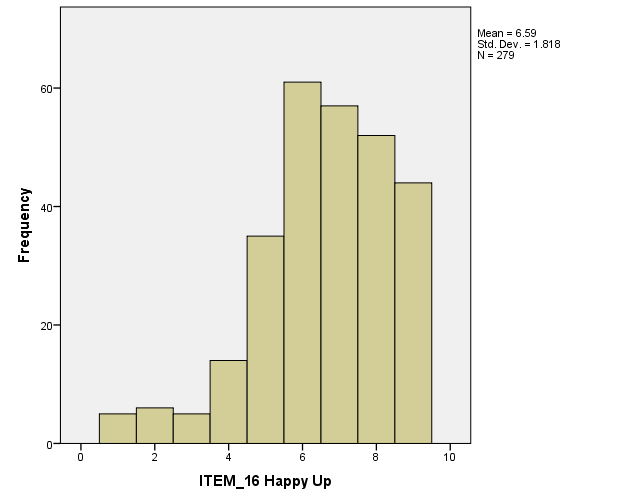

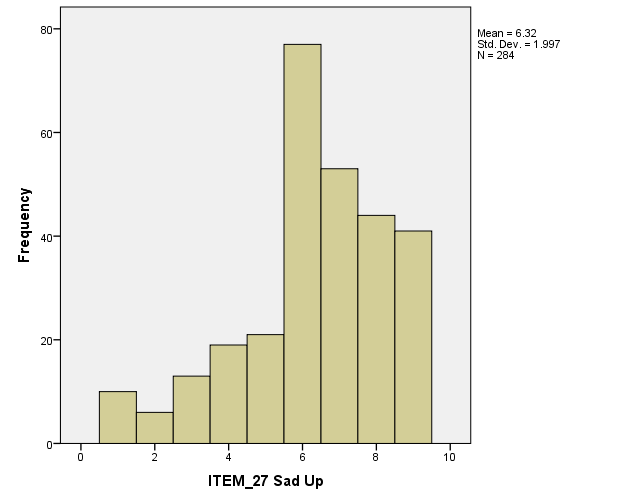


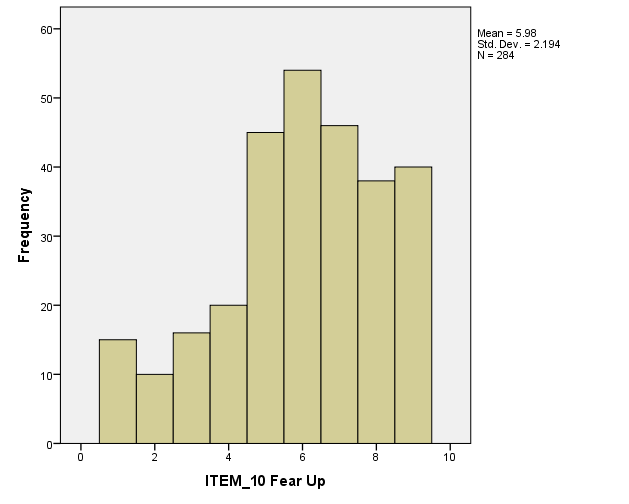

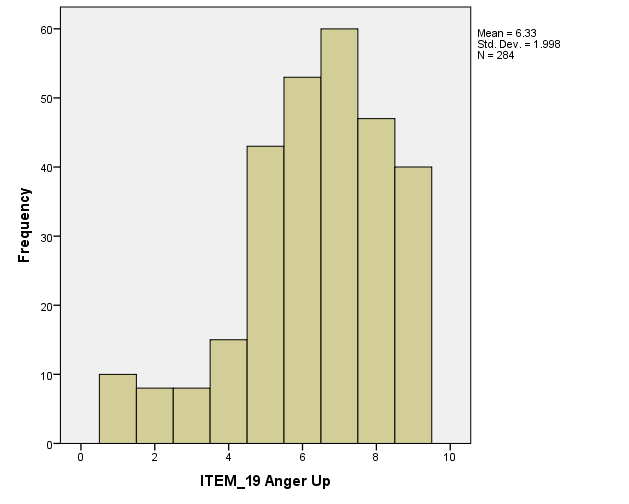


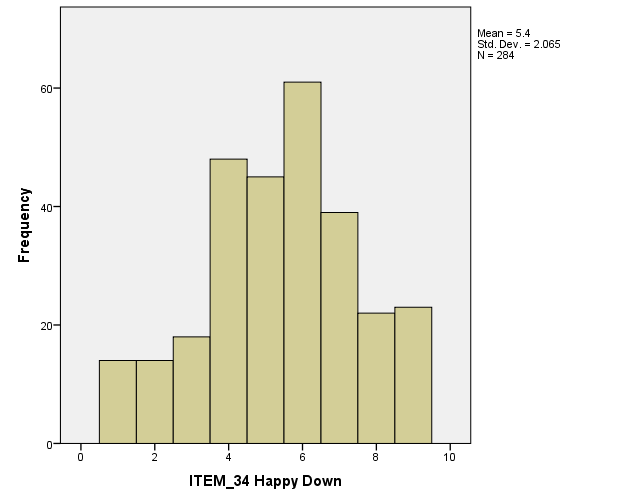

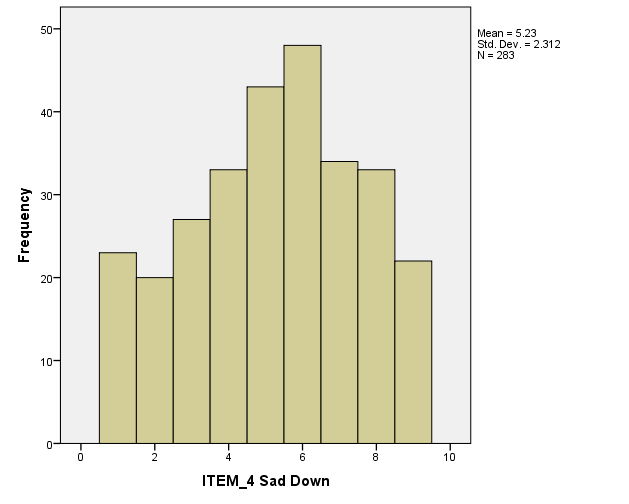


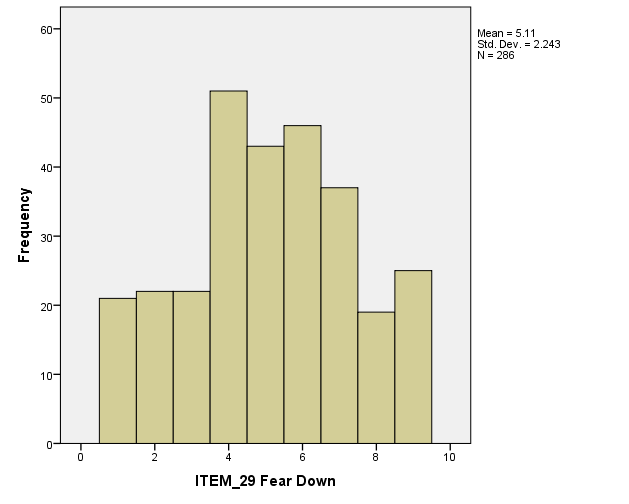

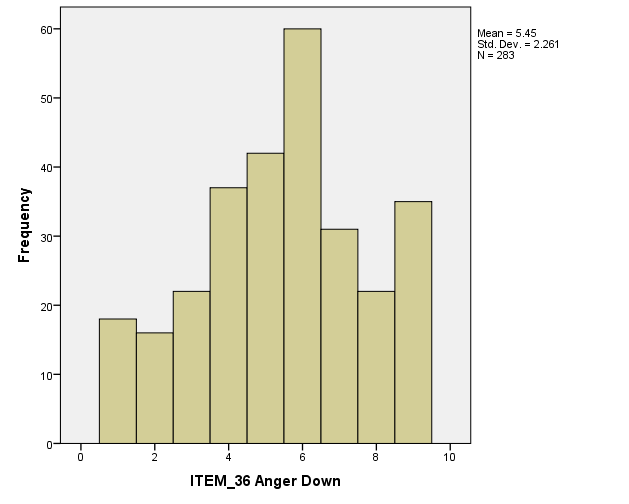


**Effect of Age**

To test whether age is associated with the perceived ability to up- and down-regulate love, an analysis of covariance (ANCOVA) with the continuous predictor Age and the factor Direction (up-regulate, down-regulate) was performed on the scores of items 1 and 2. Neither of the effects involving Age were significant, both *Fs*<2.2, both *ps*>.15.

To test whether age is associated with the perceived ability to up- and down-regulate different love types, an ANCOVA with the continuous predictor Age and the factors Direction (up-regulate, down-regulate) and Love Type (infatuation, attachment, sexual desire) was performed on the scores of items 3-8. None of the effects involving Age were significant, all *F*s<3.2, *ps*>.050.

To test whether age is associated with the perceived ability to exaggerate and suppress different love types, an ANCOVA with the continuous predictor Age and the factors Direction (exaggerate, suppress) and Love (infatuation, attachment, sexual desire) was performed on the scores of items 9-14. None of the effects involving Age were significant, all *Fs*<1.2, all *ps*>.33.

To test whether age is associated with the perceived ability to start and stop different love types, an ANCOVA with the continuous predictor Age and the factors Direction (start, stop) and Love (infatuation, attachment, sexual desire) was performed on the scores of items 15-20. There was an Age x Direction interaction, *F*(1,277)=4.1, *p*=.04, *η^2^*=.015, but none of the follow-up tests were significant.

To test whether age is associated with the perceived ability to up- and down-regulate love types and emotions, an ANCOVA with the continuous predictor Age and the factors Direction (up-regulate, down-regulate) and Feeling (infatuation, attachment, sexual desire, happiness, sadness, anger, fear) was performed on the scores of items 3-8 and 21-28. There was an Age x Direction x Feelings interaction, *F*(6,1500)=3.0, *p*=.012, *η^2^*=.012, but none of the follow-up tests were significant.

To conclude, we found no evidence that age is associated with perceived ability up- and down-regulate love, up- and down-regulate different love types, exaggerate and suppress different love types, start and stop different love types, and up- and down-regulate infatuation, attachment, sexual desire, happiness, sadness, fear, and anger.
